# Upregulation of circFLNA contributes to laryngeal squamous cell carcinoma migration by circFLNA–miR-486-3p-FLNA axis

**DOI:** 10.1186/s12935-019-0924-9

**Published:** 2019-07-29

**Authors:** Jian-Xing Wang, Yan Liu, Xin-Ju Jia, Shu-Xia Liu, Jin-Hui Dong, Xiu-Min Ren, Ou Xu, Hai-Zhong Zhang, Hui-Jun Duan, Chun-Guang Shan

**Affiliations:** 1 Department of Pathology, Hebei Medical University, 361 Zhongshan East Road, Shijiazhuang, 050017 People’s Republic of China; 2 Department of Otolaryngology, The Second Hospital of Hebei Medical University, 215 Heping West Road, Shijiazhuang, 050000 People’s Republic of China; 3 Department of Anesthesiology, The 4th Hospital of Hebei Medical University, 169 Tianshan Street, 050000 Shijiazhuang, People’s Republic of China; 4 Departmen of Endocrinology, The First Hospital of Hebei Medical University, 89 Donggang Road, Shijiazhuang, 050000 People’s Republic of China

**Keywords:** Laryngeal squamous cell carcinoma, FLNA, circRNAs, Migration, miRNA

## Abstract

**Background:**

Accumulating evidence shows that circular RNAs (circRNAs) plays vital roles in tumor progression. However, the biological functions of circRNAs in laryngeal squamous cell carcinoma (LSCC) metastasis is still unclear.

**Methods:**

qRT-PCR was used to detect circFLNA, miRNAs and FLNA mRNA expression. Transwell assay and western blot were performed to evaluate cell migration ability and to detect FLNA, MMP2 and MLK1 protein expression, respectively. RNA pull-down analysis was used to find the binding-miRNAs of circFLNA. Luciferase reporter assay was used to examine the effect of circFLNA on miRNAs and miR-486-3p on FLNA expression.

**Results:**

In this study, we confirmed that a Filamin A (FLNA)-derived hsa_circ_0092012 known as circFLNA, was upregulated in LSCC, and the higher expression of circFLNA was correlated with LSCC lymph node metastasis. Increased circFLNA facilitates LSCC cell migration ability through upregulating FLNA and MMP2 protein expression. Mechanistically, we find that circFLNA sponges miR-486-3p in LSCC cells, relieving miR-486-3p-induced repression of FLNA which promotes LSCC cell migration. Accordingly, FLNA mRNA is overexpressed in LSCC tissues and a higher FLNA level is correlated with poor survival. Dysregulation of the circFLNA/miR-486-3p/FLNA regulatory pathway contributes to LSCC migration.

**Conclusions:**

In summary, our study sheds light on the regulatory mechanism of circFLNA in LSCC migration via sponging miR‐486-3p, which downregulates the FLNA protein expression. Targeting circFLNA/miR-486-3p/FLAN axis provides a potential therapeutic target for aggressive LSCC.

## Background

Laryngeal carcinoma is the second most frequently diagnosed head and neck cancer worldwide [1], with an estimated 17,590 new cases and 3230 deaths projected to occur in the United States in 2018 [2]. Laryngeal squamous cell carcinoma (LSCC) is the most common type of laryngeal carcinoma and accounts for ~ 95% of laryngeal carcinoma cases. Currently, the main treatment for LSCC is surgery, followed by radiotherapy and chemotherapy [3, 4]. Disease specific survival rates for limited cancers (stages I, II) typically range from 60 to 90%. However, once recurrence or distant metastasis occours, patients remains worse 5-year survival rate of 64% and almost die of this disease [5, 6]. Therefore, there is an urgent need to study the underlying molecular mechanisms that contribute to LSCC migration.

Circular RNAs (circRNAs) are a new class of endogenous non-coding RNAs [7]. Unlike linear RNAs containing a 5′ caps and 3′ tails, circRNAs are characterized by covalently closed-loop structures and therefore do not have 5′ and 3′ ends [8]. Recently, increasing evidence has confirmed that circRNAs play critical roles in carcinogenesis and cancer progression [9], including proliferation, apoptosis, migration and invasion. For example, circHIPK3 derived from exon 2 of the HIPK3, regulates hepatocellular carcinoma proliferation and migration [10]; circITCH acts as a tumor suppressor contributing to bladder cancer proliferation [11]; and circRNA_104916 regulates colon cancer cell apoptosis, migration, and invasion [12]. A previous study has found that 302 circRNAs are upregulated, while 396 are downregulated in LSCC tissues compared with that in non-tumor tissues as per a microarray analysis [13]. However, the biological functions of circRNAs in LSCC metastasis remain unclear.

A previous study demonstrated that the expression of hsa_circ_0092012, termed circFLNA form exon-9 to exon-15 of the filamin A (FLNA) gene, is upregulated in human LSCC via high-throughput circRNA microarray [13]. Nevertheless, the role of circFLNA in LSCC remains unknown. In the present study, we first confirmed that circFLNA was present and upregulated in LSCC tissues and cell lines. A functional study showed that the overexpression of circFLNA promoted LSCC cell migration. Mechanistically, circFLNA sponged miR-486-3p in LSCC cells, relieving the miR-486-3p effect, leading to elevation of migration-related FLNA expression thus affecting LSCC migration and progression.

## Methods

### Tissues collection

All 39 pairs of LSCC tissues and adjacent normal epithelial tissues were obtained from patients who had undergone surgery and received primary surgical resection of LSCC between September 2017 and July 2018 in the Department of Otolaryngology, Second Hospital of Hebei Medical University. None of patients with LSCC were treated with radiotherapy or chemotherapy prior to surgery. All patients with LSCC were histopathologically and clinically diagnosed. The patients were further divided into two groups according to lymph node metastasis. The study protocol was approved by the Ethics Committee of Second Hospital of Hebei Medical University and written consent was obtained from each patient (HebMU20080026). All of experiments in this paper obey World Medical Association Declaration of Helsinki.

### Cell culture condition and transfection

Three human LSCC cell lines (Tu212, SCC-2 and SCC-40) were purchased from American Type Culture Collection (Manassas, VA, USA). A normal human keratinocyte cell line (HOK), and LSCC cell line Hep2 were available in our own lab. All cells were maintained in RPMI-1640 (Gibco, Beijing, China) with 10% fetal bovine serum (FBS) (Clark Bio, Claymont, DE, USA), 100 U/ml penicillin and 100 μg/ml streptomycin. According to the manufacturer’s protocol, the transfection was carried out using Lipofectamine 2000 (Invitrogen). The miR-486-3p mimic, mimic-negative control (NC), miR-486-3p inhibitor, inhibitor-NC, miR-574-5p mimic, miR-1275 mimic, sh-FLNA, sh-circFLNA and shCtl were purchased from GenePharma Co., Ltd (Shanghai, China). The sequences of sh-RNA were shown in follows: sh-circFLNA-1#: GUGCCAGCUCCCUGAAGGGTT; si-circFLNA-2#: GCCAGCUCCCUGAAGGGGCTT; sh-FLNA-1#: CCGCCAAUAACGACAAGAATT; sh-FLNA-2 #: CAGGCAACAUGGUGAAGAATT. Overexpression vector and luciferase reporter vector were purchased from GENEWIZ Company (Suzhou, China).

### Reverse transcription-quantitative polymerase chain reaction (RT-qPCR) analysis

Total RNA was extracted using QIAzol Lysis Reagent (Qiagen, 79306) according to the manufacture’s protocol. The concentration and purity of total RNA were measured using a NanoDrop^®^ spectrophotometer (NanoDrop; Thermo Fisher Scientific, Inc., Wilmington, DE, USA). For miRNA, the miScripIIRT kit (QIAGEN GmbH, D-40724 Hilden, GERMANY) was used for reverse transcription, and the miScript SYBR^®^ Green PCR kit was used for qRT-PCR according to the manufacturer’s protocol. For circRNA and mRNA expression, the M-MLV First Strand Kit (Life Technologies) was used to synthesize to cDNA from RNA. The Platinum SYBR Green qPCR Super Mix UDG Kit (Invitrogen) was used for the qRT-PCR. qPCR was carried out using Platinum SYBR Green qPCR Super Mix UDG Kit (Invitrogen). U6 and GAPDH were used as control, respectively. The primers were designed as follows: miR-34a-5p:GGCTGGCAGTGTCTTAGCTGGTTG; miR-92b-5p:AGGGACGGGACGCGGTGC; miR-296-3p:GCGAGGGTTGGGTGGAGGCTC; miR-486-3p:GCCGGGGCAGCTCAGTACAG; miR-661:TGCCTGGGTCTCTGGCCTGC; miR-574-5p:GGCTGAGTGTGTGTGTGTGAGTGTG; miR-760:CGGCTCTGGGTCTGTGGGG; miR-486-3p:GGGGCTGGGGCCGGGGCC; miR-1226-5p:GTGAGGGCATGCAGGCCTG; miR-1271-3p:AGTGCCTGCTATGTGCCAGGC; miR-1275:GGCGTGGGGGAGAGGCTGTC; miR-1287-5p:GGCTGCTGGATCAGTGGTTCGAG; FLNA-F:AATGTGACGACAAGGGCGAC, FLNA-R:AGCACGTGAACGGCATACTC; circFLNA-F:CCAGCTGAGGCTCTACCGTGCC, circFLNA-R:GAGGCGTCAGCATCCCCAACAG, linearFLNA; F:GCTTGGCCAACAGTGACAGTGTAGG, linearFLNA-R:CAGCTACCAGCCCACCATGGAG. All data were analyzed by adopting 2-ΔΔCt method as described previously [14].

### RNA pull-down analysis

RNA pull-down analysis was performed as described previously [15]. Briefly, the Hep2 cells were incubated with biotin (Bio)-labeled oligonucleotide probes against circFLNA (Bio-5′-CAACAGCCCCTTCAGGGAGCTGGCACGGGC (GenePharma Co., Shanghai, China) at 37 °C for 4 h. M-280 Streptavidin Dynabeads (Life Technologies) were added per 100 pmol of biotin-DNA oligos, and the mixture was then rotated for 30 min at 37 °C. The beads were captured by magnets (Life Technologies) and washed five times. Each experiment was replicated in triplicate.

### Western blot analysis

The cultured cells were lysed with lysis buffer. Equal amounts of protein were run on 10% SDS-PAGE, and electro-transferred to a polyvinylidene fluoride (PVDF) membranes (Millipore). Following blocking in 5% nonfat milk, the membranes were incubated with special primary antibodies as follows: anti-MKL1 (1:1000, ab49311), anti-MMP2 (1:1000, ab37150), anti-FLNA (1:1000, ab51217) and anti-β-actin (1:1000, sc-47778). The blots were treated with the Immobilon™ Western (Millipore), and detected by ECL (enhanced chemiluminescence) Fuazon Fx (Vilber Lourmat). Images were captured and processed using FusionCapt Advance Fx5 software (Vilber Lourmat). All experiments were replicated in triplicate.

### Luciferase assay

Luciferase assay was performed as previously described [15]. For circFLNA-binding-miRNA luciferase assays, the Hep2 cells were co-transfected with an miRNA mimic (Gene pharma; Shanghai) or NC mimic (200 pmol) combined with 100 ng of circFLNA-luciferase reporter or an empty vector; For the miR-486-3p-FLNA luciferase assay, the Hep2 cells were co-transfected with a miRNA-486-3p mimic or NC mimic combined with FLNA-3′UTR-luciferase reporter (wt or mut). Dual-Glo Luciferase Assay system (Promega, Madison, WI) was used to detected luciferase activity according to the manufacturer’s protocols. Firefly luciferase activity was measured and normalized against the Renilla luciferase (RLuc) activity.

### Transwell migration assay

Cell migration ability was tested by 8-μm pore size transwell filiters (Costar, Cambridge, Massachusetts). In brief, Hep2 cells (1 × 105 cells/well) were transferred onto the upper chambers of a serum-free culture. RPMI-1640 containing 10% FBS was added to the lower chambers. Following incubation at 37 °C and 5% CO_2_ for 24 h, migratory cells on the upper side of the chamber and medium part of the lower chamber was scraped off with a cotton swab. Then the membranes were stained by crystal violet solution. The migratory cell number was counted in three randomly areas using a microscope.

### Target prediction

miRanda (http://www.microrna.org), RNAhybrid (http://bibiserv.techfak.uni-bielefeld.de/rnahybrid/submission.html) RNA22 (https://cm.jefferson.edu/rna22/Interactive/) were used to identify the potential target miRNAs of circFLNA; Targetscan (http://www.targetscan.org) was used to identify the potential target gene of miRNA.

### Statistical analysis

Data were presented as mean ± SEM. Student’s *t* test was used to analyze differences between two groups. Spearman’s correlation analysis was use to evaluate the correlation analysis. Values of *P *< 0.05 were considered statistically significant. Graphpad Prism 7.0 software was using to perform the statistical analysis (GraphPad Software, San Diego, CA, USA).

## Results

### circFLNA is upregulated in LSCC tissues and correlates with lymph node metastasis

**Fig. 1:**
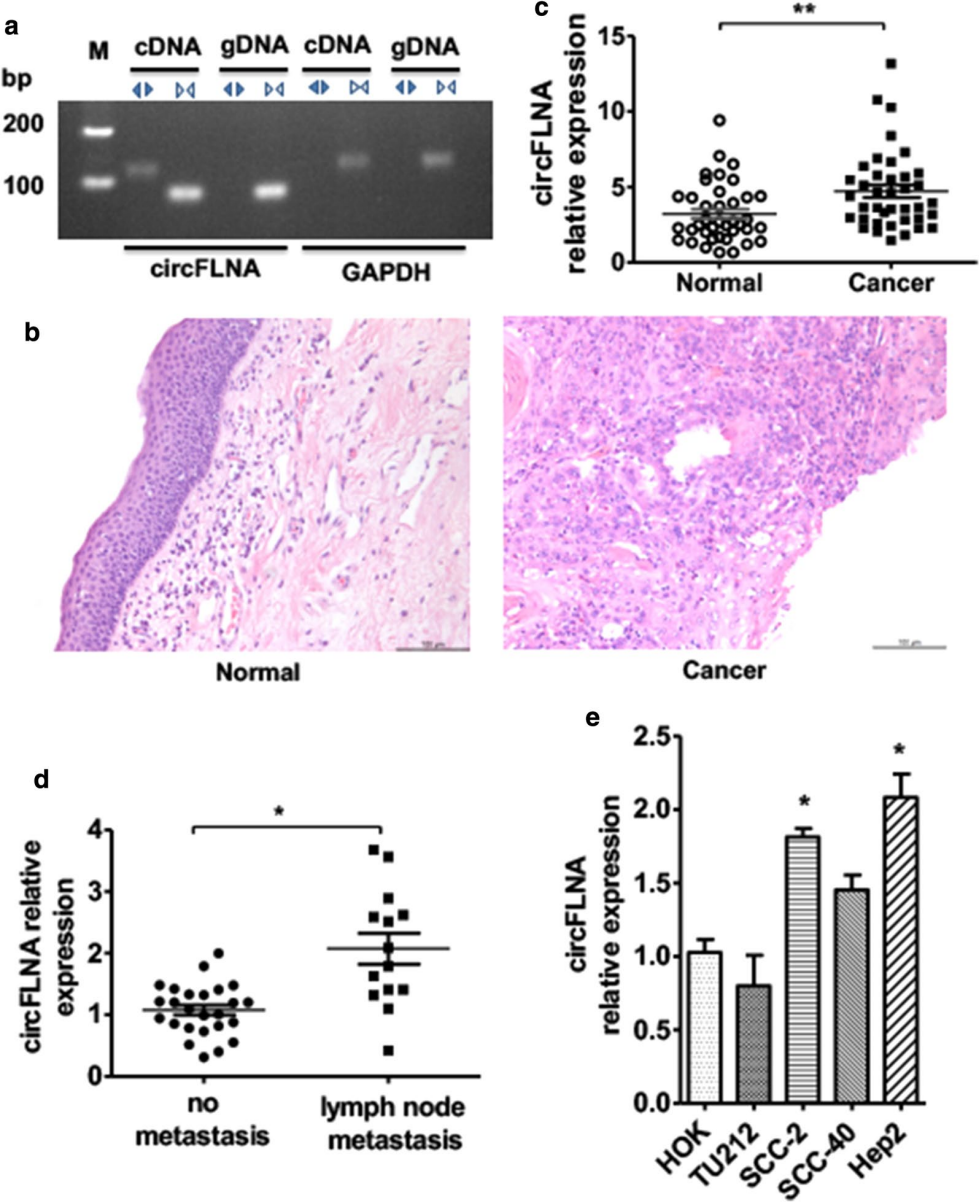
circFLNA is upregulated in LSCC tissues and correlates with lymph node metastasis. **a** PCR was used to detect circFLNA in LSCC tissues by using convergent or divergent primers. Divergent primers amplify circFLNA in cDNA but not in genomic DNA (gDNA). GAPDH was used as linear control. **b** Hematoxylin and eosin staining of normal laryngeal tissues and LSCC tissues. **c** circFLNA expression in LSCC tissues (n = 39) and adjacent normal tissues (n = 39) detected by qRT-PCR analysis. Normalized to GAPDH. **P < 0.01 vs. adjacent normal tissues. **d** LSCC patients divided into two groups, with lymph node metastasis group or without lymph node metastasis group. qRT-PCR detected the expression of circFLNA in two groups. *P < 0.05 vs. group without lymph node metastasis. **e** qRT-PCR analysis detected the expression of circFLNA in four LSCC cell lines (SCC-2, SCC-20, Hep2 and Tu212) compared with human oral keratinocyte cell line HOK. *P < 0.05 vs. HOK cell

To identify the biofunctions of circFLNA in LSCC progression, we first used divergent primers to amplify the circRNAs formed by head-to-tail splicing. The agarose gel of PCR products confirmed that circFLNA was present in the LSCC tissues (Fig. 1a). We then utilized qRT-PCR to detect the expression of circFLNA in LSCC (n = 39) tissues and adjacent normal tissues (n = 39). The results showed that the circFLNA level was higher in LSCC tissues compared with that in adjacent normal tissues (Fig. 1b, c). Next, the 39 LSCC patients were classified into two groups according to the presence or absence of lymph node metastasis. Surprisingly, circFLNA expression was higher in the group with lymph node metastasis (n = 14) than in that without lymph node metastasis (n = 25) (Fig. 1d). In order to investigate the clinical significance of circFLNA in LSCC, we analyzed the circFLNA expression level in 39 LSCC and their clinicopathologic characteristics. The correlation analysis of circFLNA expression significantly associated with lymph node metastasis (*P *= 0.048) (Table 1). However, there was no significant correlation between circFLNA expression and other clinicopathologic factors, such as age, smoking, sex, T classification, clinical stage and pathological grade. Moreover, RT-qPCR was used to detect the circFLNA expression in four LSCC cell lines (SCC-2, SCC-40, Hep2 and Tu212) as compared with that in the human oral keratinocyte cell line HOK. As Fig. 1e showed, circFLNA expression was elevated in Hep2 and SCC-2 cells but not in the SCC-40 or Tu212 cells. These findings suggest that circFLNA was upregulated in LSCC tissues and may be correlated with tumor metastasis.

**Table 1:** Correlation between circFLNA mRNA expression and clinicopathological characteristics

Characteristics	Number of patients (%)	circFLNA expression		*P* value^a^
		Low (%)	High (%)	
Total no. of patients	39	20	19	
Age (years)				
≤ 60^b^	18	10 (66.67)	8 (33.33)	0.751
> 60	21	10 (47.62)	11 (52.38)	
Gender				
Male	31	15 (48.39)	16 (51.61)	0.695
Female	8	5 (62.50)	3 (37.50)	
T stage				
T1/2	16	7 (43.75)	9 (56.25)	0.523
T3/4	23	13 (56.52)	10 (43.48)	
Lymph node metastasis				
N0	25	16 (64.00)	9 (36.00)	*0.048*
N+	14	4 (28.57)	10 (71.43)	
Clinical stage				
I/II	15	6 (40.00)	9 (60.00)	0.333
III/IV	24	14 (58.33)	10 (41.67)	
Pathological grade				
G1/2	27	15 (55.56)	12 (44.44)	0.501
G3	12	5 (41.67)	7 (58.33)	
Smoking status				
Non-smoker	7	3 (42.86)	4 (57.14)	0.695
Smoker	32	17 (53.13)	15 (46.87)	
Alcohol consumption				
Non-drinker	8	3 (37.50)	5 (62.50)	0.451
Drinker	31	17 (54.84)	14 (45.16)	

Significant associations are shown in italic face in the P value column (*P* value < 0.05)

^a^Chi square test

^b^Median age

### circFLNA plays an essential role in LSCC cell migration

**Fig. 2:**
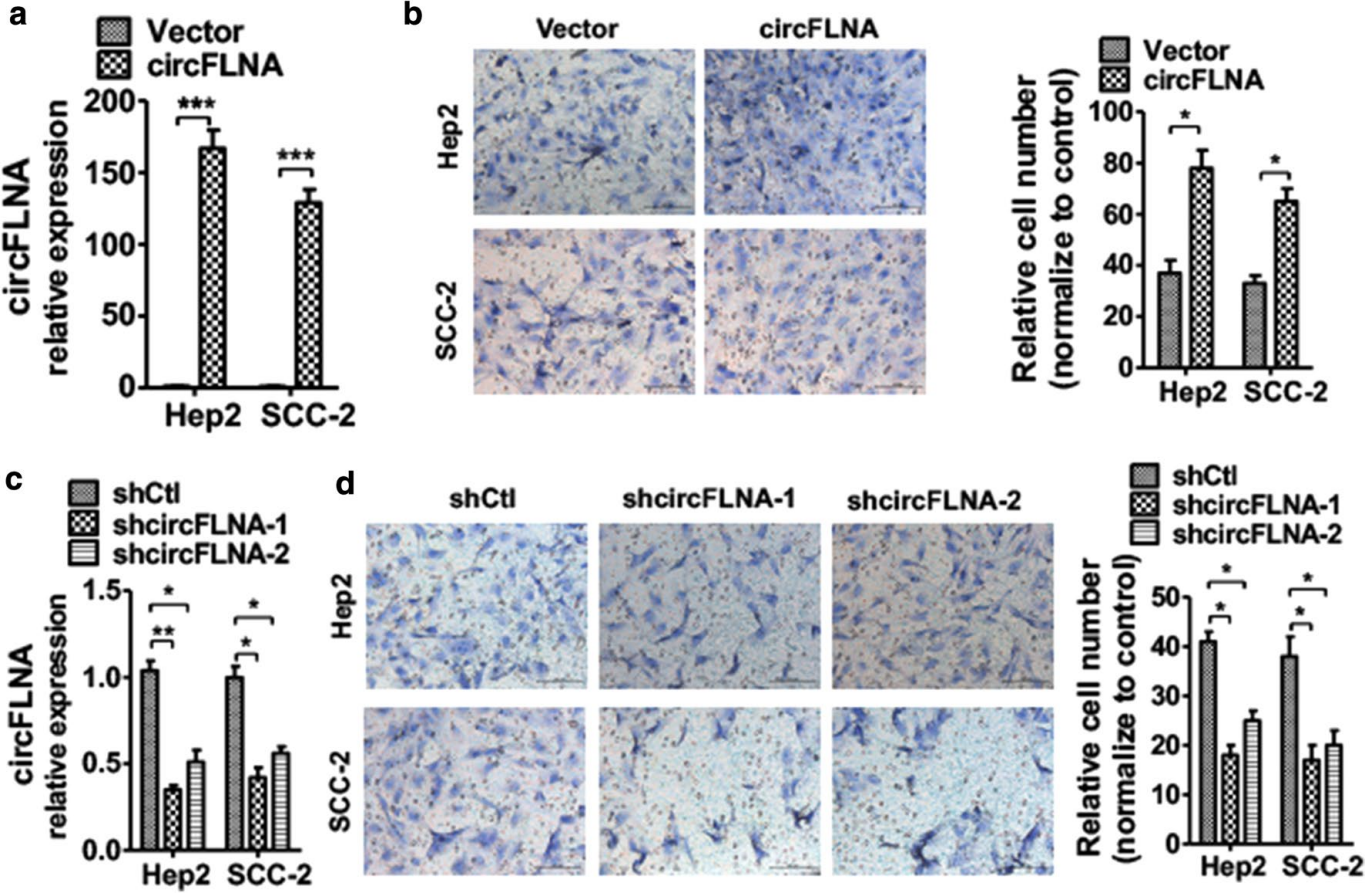
circFLNA plays an essential role in LSCC cell migration. **a** Hep2 cells were transfected with circFLNA overexpression vector pcDNA3.1–circFLNA or empty vector. circFLNA level was detected by qRT-PCR analysis. ***P < 0.001 vs. empty vector. **b** Hep2 cells were treated as (**a**), cell migration was detected by transwell assay. Right panel shows migration cell number analysis of three independent experiments. *P < 0.05 vs. empty vector. **c** Hep2 cells were transfected with shcircFLNA-1, shcircFLNA-2, or negative control (shCtl). qRT-PCR were used to examine circFLNA level. *P < 0.05 vs. shCtl. **d** Hep2 were treated as **c**, cell migration was detected by transwell assay. Right panel shows migration cell number. *P < 0.05 vs. shCtl

Because of circFLNA was upregulated in LSCC tissues with lymph node metastasis, we performed a loss-and-gain experiment to investigate whether circFLNA was a response to LSCC cell migration. First, Hep2 and SCC-2 cells were transfected with circFLNA overexpression plasmid, pcDNA3.1-circFLNA or its empty vector. As shown in Fig. 2a, transfection with pcDNA3.1-circFLNA markedly increased circFLNA level in Hep2 and SCC-2 cells. Then, transwell migration assays showed that the overexpression of circFLNA significantly promoted Hep2 cell and SCC-2 cell migration abilities (Fig. 2b). In contrast, LSCC cells were transfected with shRNA against circFLNA or its negative control. RT-qPCR analysis showed that shcircFLNA transfection caused considerable downregulation of circFLNA level in Hep2 cell and SCC-2 cells as compared with that of control shRNA. Meanwhile, transwell migration assays revealed that knockdown of circFLNA inhibited Hep2 cell migration. These results suggest that overexpression of circFLNA promotes LSCC cell migration.

### circFLNA promotes cell migration by upregulating FLNA protein expression

**Fig. 3:**
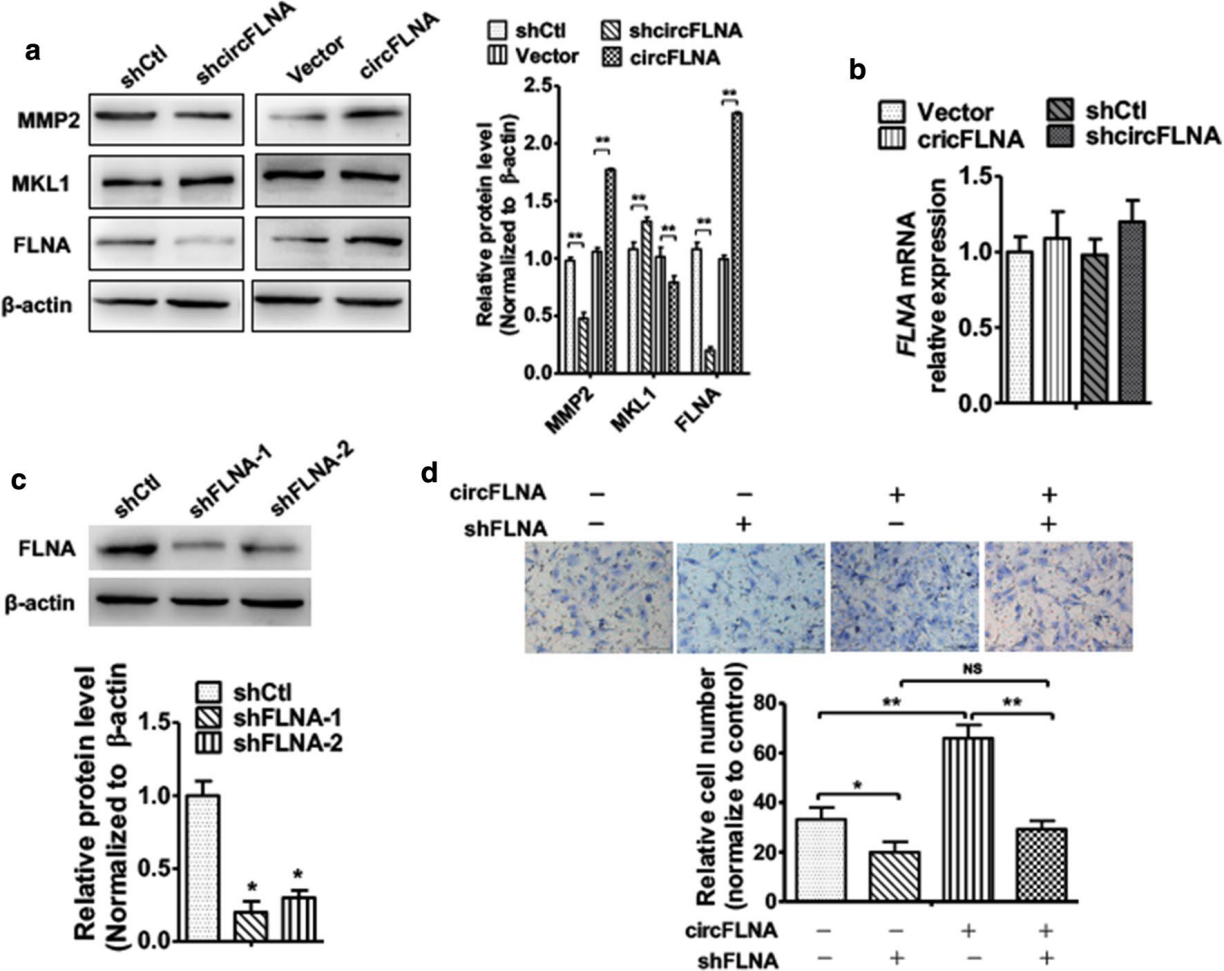
circFLNA promotes cell migration by upregulating FLNA protein expression. **a** Hep2 cells were transfected pcDNA3.1–circFLNA or pcDNA3.1, shFLNA or negative control, respectively. Western blot analysis was used to detect MMP2 and FLNA protein level. β-actin was used as an internal control. Right panel shows densitometric analysis. **P < 0.01 vs. empty vector. **b** Hep2 cells were prepared as **a**, qRT-PCR were used to detect FLNA mRNA expression. **c** Hep2 cells were transfected shFLNA or negative control. Western blot analysis were used to examine FLNA protein level. Bottom panel shows densitometric analysis. *P < 0.05 vs. empty vector. **d** Hep2 cells were transfected with pcDNA3.1-circFLNA or sh-FLNA, respectively, or co-transfected with both together. Cell migration was detected by transwell assay. Bottom panel shows migration cell number. *P < 0.05, **P < 0.01 vs. corresponding control

**Fig. 4:**
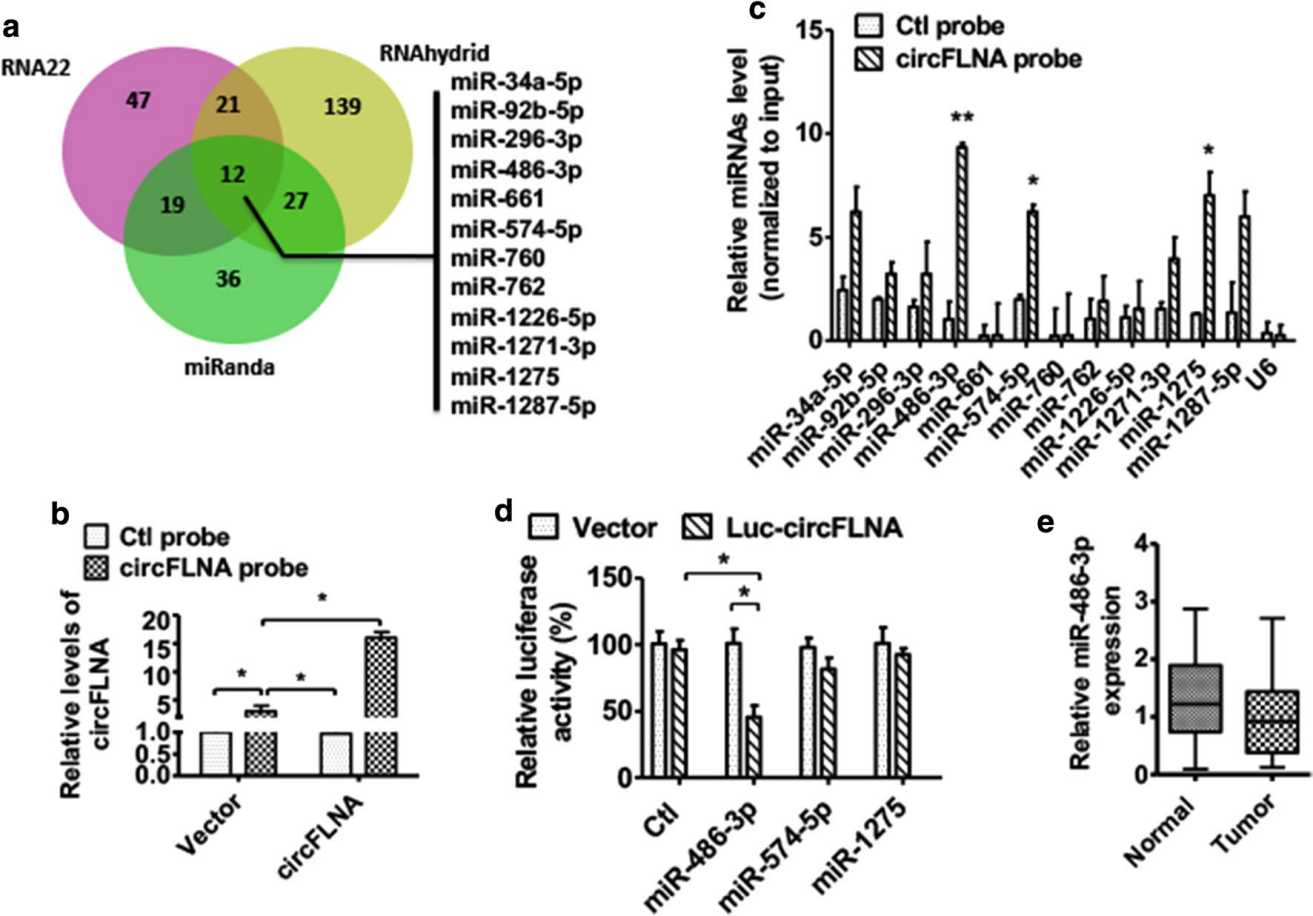
circFLNA functions as a sponge of miR-486-3p in LSCC cell. **a** Venn diagram hybrid of potential binding miRNAs from three different target prediction programs. **b** Hep2 cells were transfected with pcDNA3.1-circFLNA or empty vector, pulled down from Hep2 cell lysates with biotin-labeled circFLNA or Ctl probe, qRT-PCR detected pulldown efficiency. *P < 0.05 vs. corresponding control. **c** The miRNAs were pulled down from Hep2 cell lysates with biotin-labeled circFLNA or Ctl probe. qRT-PCR detected the relative expression of indicated miRNAs. Normalized to U6. *P < 0.05, **P < 0.01 vs. NC probe. **d** Luciferase reporter assays were performed in Hep2 cells co-transfected with wild-type (WT) or mutant (mut) circFLNA-luciferase reporter and miRNA mimics. *P < 0.05 vs. empty vector. **e** The expression of miR-486-3p in LSCC tissues and adjacent normal tissues was detected by qRT-PCR analysis

Because of FLNA is considered as an important oncogene in multiple types of cancer owing to its role in cancer cell proliferation, apoptosis and migration [16]. We next examined whether circFLNA-associated migration by regulating FLNA expression. As shown in Fig. 3a, overexpression of circFLNA substantially increased the migration-marker protein matrix metalloproteinase-2 (MMP-2) level and FLNA protein level; however, it did not affect megakaryoblastic leukemia 1 (MKL1) protein expression in the Hep2 cells. In contrast, knockdown of circFLNA in Hep2 cell markedly decreased MMP-2 and FLNA protein level (Fig. 3a). Subsequently, RT-qPCR analysis showed that overexpression or knockdown of circFLNA expression in Hep2 cells did not affect FLNA mRNA expression (Fig. 3b). In order to confirm the function of FLNA in LSCC migration, we then knocked down the FLNA level in Hep2 cells. Transfection of shFLNA in Hep2 cells significantly decreased the FLNA protein level compared with that of control shRNA-transfected cells (Fig. 3c). Depletion of FLNA in Hep2 cells decreased Hep2 cell migration (Fig. 4d line 2) compared with negative control. Moreover, the migration ability was partly rescued following the overexpression of FLNA compared with that following the overexpression of circFLNA alone (Fig. 4d). These findings indicate that circFLNA positively regulates the FLNA protein expression and is associated with LSCC cell migration.

### circFLNA sponges miR-486-3p in LSCC cell

Because of circRNAs functions as miRNAs sponges to regulate gene expression, we sought to analyze the potential binding miRNAs of circFLNA. First, we identified the miRNA-binding sites in the circFLNA sequence by using three target prediction programs, miRanda, RNA22 and Rnahdrid. As shown in Fig. 4a, circFLNA contained sequences complementary to miR-34a-5p, miR-92b-5p, miR-296-3p, miR-486-3p, miR-661, miR-574-5p, miR-760, miR-486-3p, miR-1226-5p, miR-1271-3p, miR-1275 and miR-1287-5p. Second, biotin-labeled circFLNA probe pull-down assay was used to examine the expression of miRNAs in the circFLNA-overexpressed Hep2 cells. Using RT-qPCR analysis, the pull-down efficiency of circFLNA was obviously enhanced in circFLNA-overexpressed Hep2 cells (Fig. 4b). Third, RT-qPCR analysis was used to detect the level of the candidate miRNAs in the precipitates pulled down with biotin-labeled circFLNA. As shown in Fig. 4c, miR-486-3p, miR-547 and miR-1275-5p were significantly enriched in the circFLNA-overexpressed precipitates. Furthermore, luciferase assays showed that co-transfection with circFLNA-luciferase-reporter vector and miR-486-3p, but not miR-574-5p or miR-1275-5p, significantly decreased the luciferase activity mediated by wild-type circFLNA sequence. Neither miR-574-5p nor miR-1275-3p decreased the luciferase activity mediated by the miRNA binding site-mutated circFLNA (Fig. 4d). In addition, the expression of miR-486-3p was not significantly different between the LSCC tissues and adjacent normal tissues (Fig. 4e). These results indicate that circFLNA sponges miR‐486-3p in the LSCC cells.

### FLNA is a direct target of miR-486-3p

**Fig. 5:**
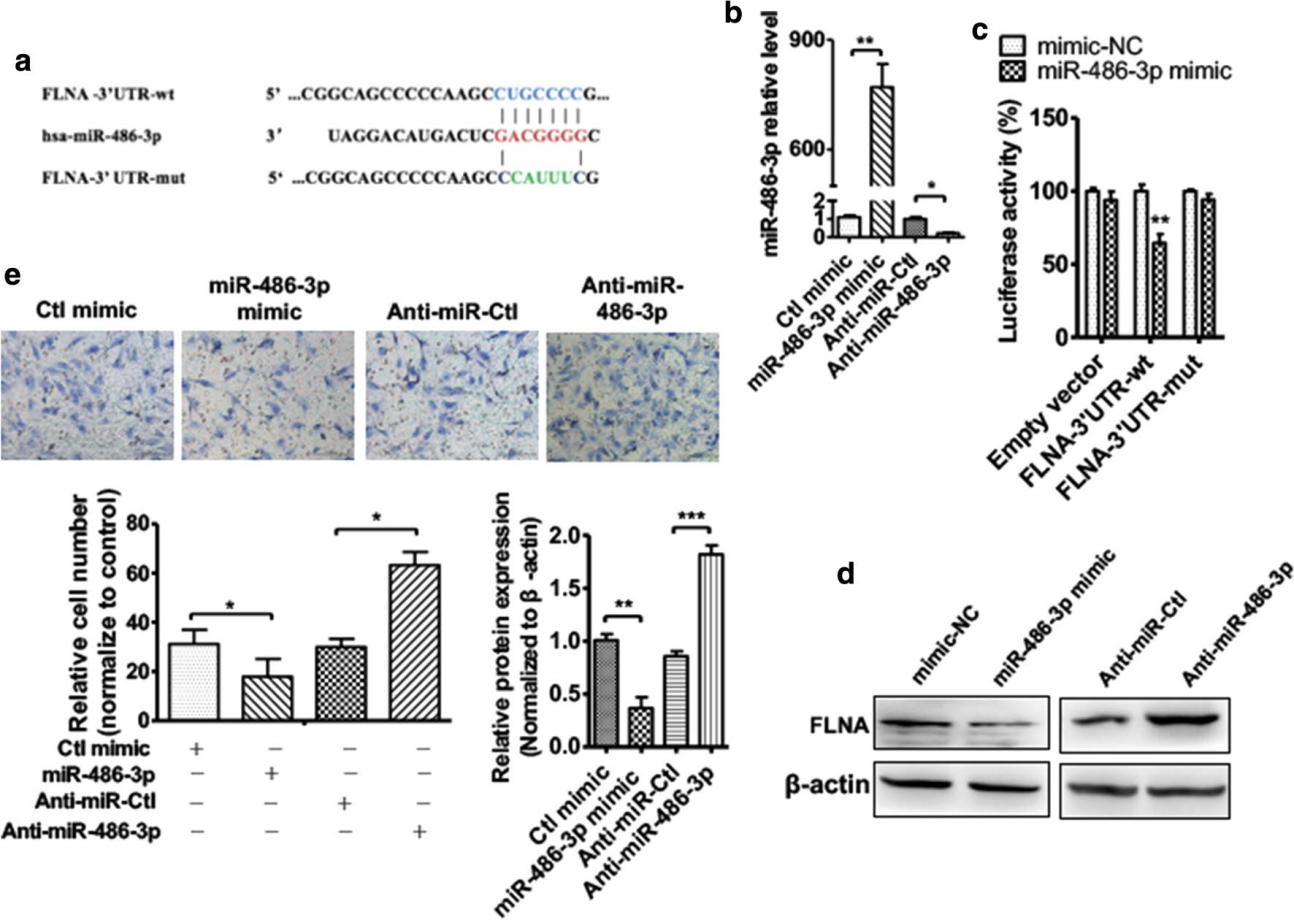
FLNA is a direct target of miR-486-3p. **a** Potential miR-486-3p binding site in FLNA 3′-UTR. **b** Hep2 cells were transfected with miR-486-3p mimic, miR-486-3p inhibitor or its correspondence control. qRT-PCR detected the level of miR-486-3p. *P < 0.05, **P < 0.01 vs. corresponding control. **c** Hep2 cells were co-transfected with miR-486-3p mimic or mimic-NC and pmir-GLO vector containing wild-type or mutated miR-486-3p-binding site (mut) at FLNA3′-UTR. Luciferase reporter assays were performed. *P < 0.05 vs. mimic-NC. **d** Hep2 cells treated as **b**, western blot analysis was used to detected FLNA protein level. Bottom panel shows densitometric analysis of three independent experiments. **P < 0.001 vs. corresponding control. **e** Hep2 cells treated as **b**, cell migration was detected by transwell assay. Right panel shows migration cell number analysis of three independent experiments. *P < 0.05 vs. corresponding control

Because of circRNAs may function as miRNA sponges and form the circRNA-miRNA-mRNA axis to play its biological effect in gene regulation, we used miRNA target prediction to identify the target genes of miR-486-3p. Surprisingly, FLNA was found to contain highly conserved miR-486-3p-binding sites in its 3′UTR (Fig. 5a). Thereafter, Hep2 cells transfected with miR-486-3p mimic or inhibitor, respectively. The transfection efficiency was detected using RT-qPCR. As shown in Fig. 5b, transfection with miR-486-3p mimic significantly increased while transfected miR-486-3p decreased the miR-486-3p level in Hep2 cells, compared with the negative control. Next, Hep2 cells were co-transfected with FLNA-3′UTR-reporter plasmid containing the miR-486-3p-binding site (wt or mut) and miR-486-3p mimic. As we expected, miR-486-3p mimic significantly decreased luciferase activity mediated by wild-type 3′-UTR but had no effect on the luciferase activity mediated by its mutant (Fig. 5c). In order to obtain further evidence supporting the function of miR-486-3p in LSCC cell migration, we transfected Hep2 cells with mimic and inhibitor or their corresponding control and detected the FLNA protein expression. As shown in Fig. 5d, depletion of miR-486-3p increased the FLNA expression level while overexpression of miR-486-3p markedly decreased the FLNA protein expression. Similarly, transwell migration assay showed that the miR-486-3p overexpression reduced Hep2 cell migration, whereas knockdown of miR-486-3p promoted Hep2 cell migration (Fig. 5e). These findings suggest that miR-486-3p directly reduces FLNA expression by target its 3′UTR and correlates with LSCC cell migration.

### Linear FLNA mRNA is up-regulated in LSCC tissues and correlated with poor prognosis

**Fig. 6:**
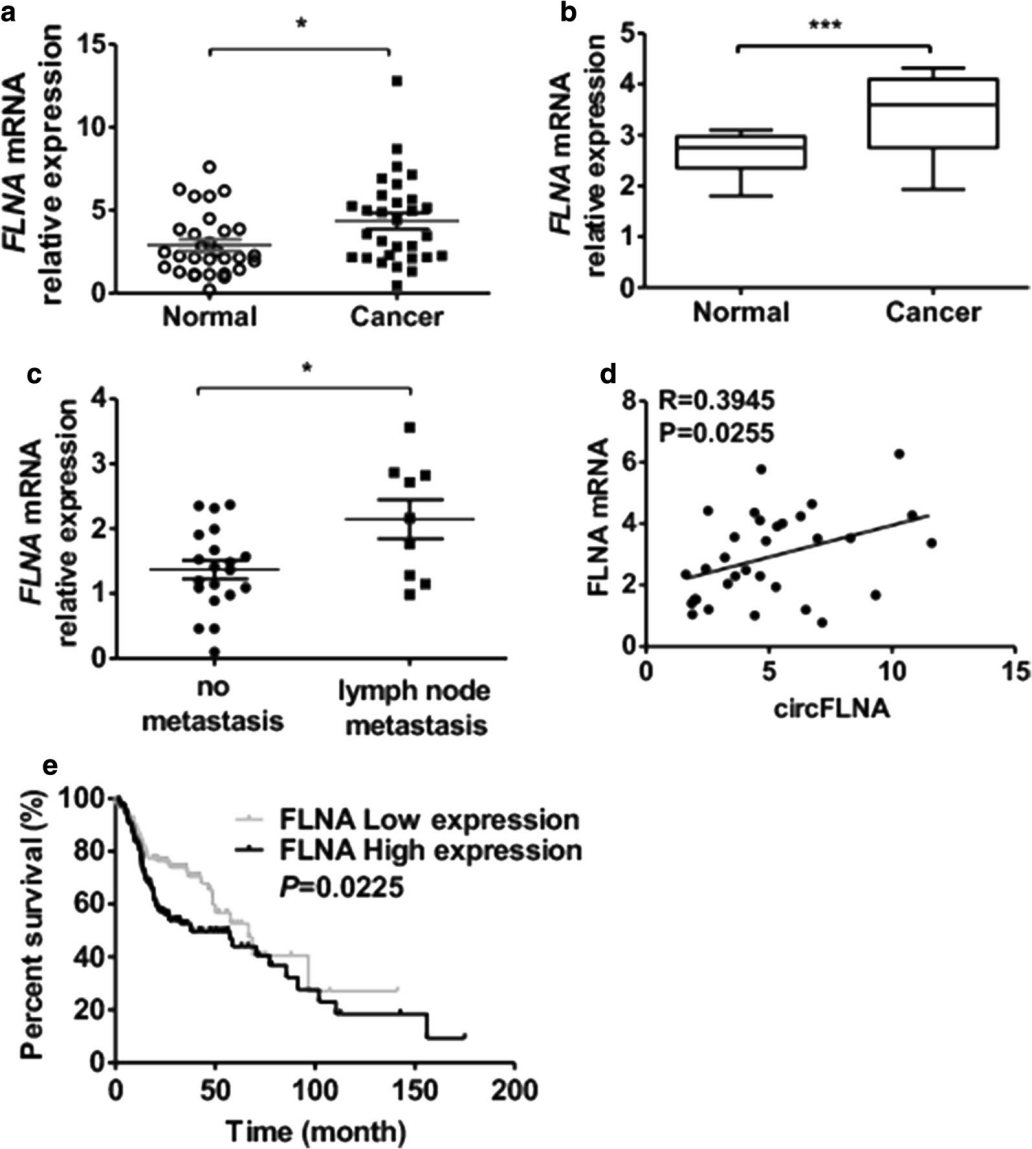
FLNA was up-regulated in LSCC tissues and correlated with poor prognosis. **a** qRT-PCR detected NPL4 mRNA expression in LSCC tissues (n = 39) and adjacent normal tissues (n = 39). *P < 0.05 vs. adjacent normal tissues. **b** FLNA mRNA expression in LSCC tumor and normal tissues from the TCGA database. **c** LSCC patients divided into two groups, with lymph node metastasis group or without lymph node metastasis group. qRT-PCR was used to detected FLNA mRNA expression in two groups. *P < 0.05 vs. group without lymph node metastasis. **d** Pearson correlation was used to analyze the relationships between FLNA mRNA and circFLNA (R = 0.3945, *P *= 0.0255). **e** NPL4 mRNA expression in Kaplan–Meier analysis of overall survival stratified by low and high FLNA expression (*P *= 0.0225)

To identify whether FLNA expression was altered in LSCC tissues, we used RT-qPCR to validate the FLNA mRNA expression in LSCC tissues and adjacent normal tissues. FLNA mRNA expression was up-regulated in LSCC tissues compared with that in adjacent normal tissues (Fig. 6a). Similar result from the TCGA database confirmed that FLNA was increased in LSCC tissues (Fig. 6b). Moreover, FLNA mRNA level was markedly higher in the lymph node metastasis group than in the group without lymph node metastasis (Fig. 6c). The correlation analysis also revealed a significant positive correlation between FLNA and circFLNA level (*P *= 0.0255, R = 0.3945) (Fig. 6d). Thereafter, using the TCGA database, data from Oncolnc (http://www.oncolnc.org/) human clinical sample surveys suggested that patients with higher FLNA mRNA expression had a significantly worse overall survival (*P *= 0.025, Fig. 6e). These findings indicate that the FLNA mRNA expression is higher in the LSCC tissues and predicts poor survival.

### circFLNA/miR-486-3p/FLNA pathway regulates LSCC cell migration

**Fig. 7:**
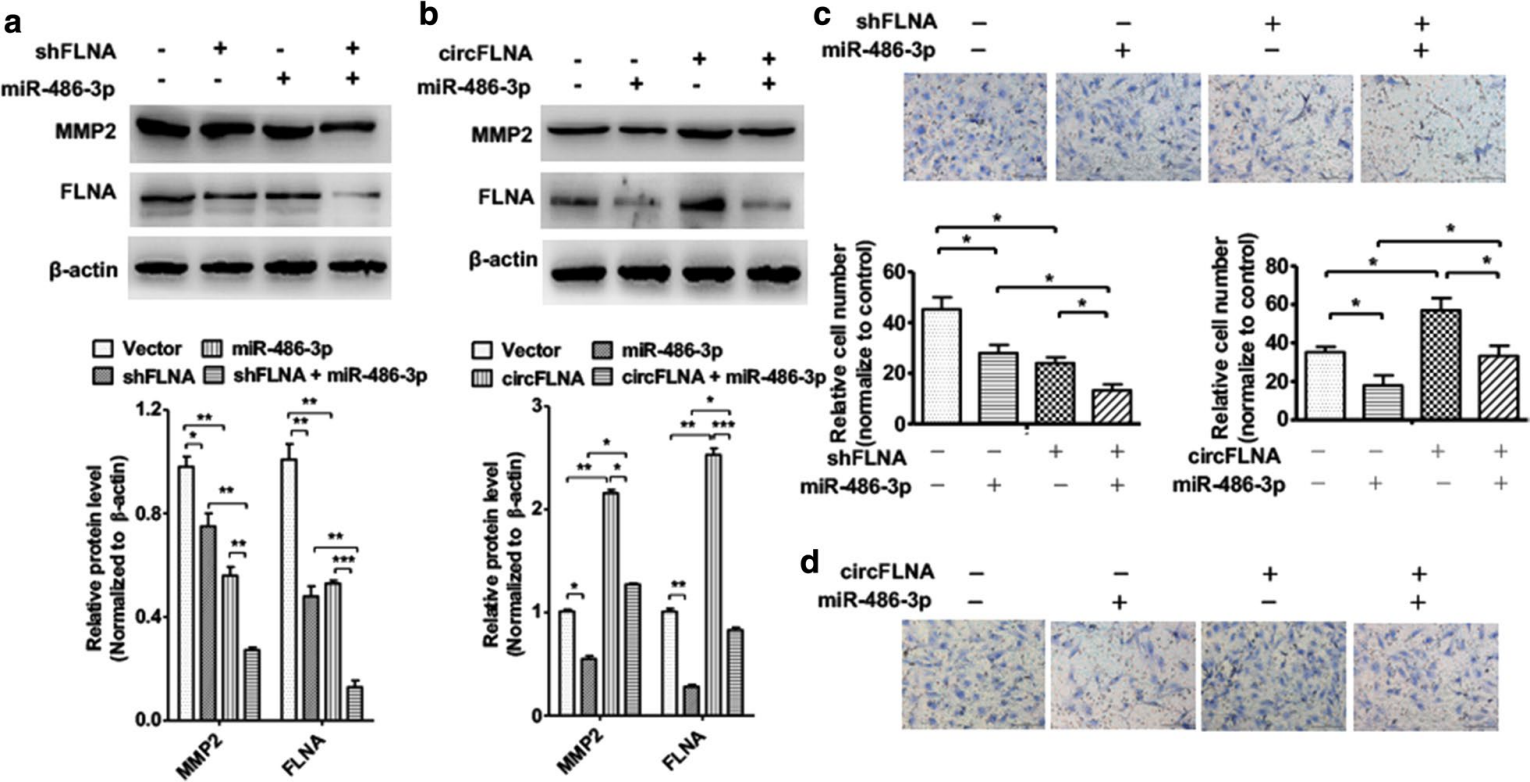
circFLNA/miR-486-3p/FLNA pathway regulates LSCC cell migration. **a** Hep2 cells were transfected with sh-FLNA or miR-486-3p mimic, respectively, or co-transfected with them together. Western blot analysis was used to determine MMP-2 and FLNA protein level. Right panel shows densitometric analysis. **P < 0.01, ***P < 0.001 vs. corresponding control. **b** Hep2 cells were treated as **a**, cell migration was evaluated by transwell assay. Bottom panel shows migration cell numbers *P < 0.05 vs. corresponding control. **c** Hep2 cells were transfected with pcDNA3.1-circFLNA or miR-486-3p mimic, respectively, or co-transfected with them together. MMP-2 and FLNA protein level was detected by western blot analysis. Right panel shows densitometric analysis.* *P *< 0.05, **P < 0.01, ***P < 0.001 vs. corresponding control. **d** Hep2 cells were prepared as **c**, cell migration was evaluated by transwell migration assay. Bottom panel shows migration cell numbers analysis of three independent experiments *P < 0.05 vs. corresponding control

In order to further investigate the relationship between the circFLNA/miR-486-3p/FLNA pathway and LSCC cell migration, some rescue experiments were performed. First, Hep2 cells were transfected with shFLNA, miR-486-3p mimic or co-transfected with both. Western blot analysis detected protein level of migration-maker MMP2. The result showed that overexpression of miR-486-3p decreased MMP-2 and FLNA protein level. Co-transfection of them together enhanced the effect of miR-486-3p-induced alone (Fig. 7a). Furthermore, transwell assays showed that knockdown of FLNA in Hep2 cells significantly inhibited cell migration compared with that in NC group and enhanced the inhibitory effect of miR-486-3p overexpression on cell migration (Fig. 7b). Constantly, Hep2 cells were transfected with pcDNA3.1-circFLNA, miR-486-3p mimic, or co-transfected with them together. As shown in Fig. 7c, overexpression of circFLNA increased expression levels of MMP2 and FLNA. However, co-transfection of miR-486-3p partly reversed circFLNA-induced MMP-2 and FLNA increase. Transwell assays also showed that overexpression of circFLNA significantly promoted cell migration compared with its negative control and reversed the inhibitory effect of miR-486-3p overexpression on cell migration (Fig. 7d). These findings further confirm that the circFLNA/miR-486-3p/FLNA axis regulates LSCC cell migration.

## Discussion

In the last few decades, several circRNAs functions have been identified, including as competing endogenous RNAs or miRNA sponges [17], interaction with RNA binding proteins [18], modulation of the stability of mRNAs [19], translation of proteins and regulation of gene transcription [20]. circRNAs has been confirmed as sponges for miRNAs that influence gene expression by reducing the inhibitory effect of miRNA on its target gene [21]. Accumulating evidence suggests that circRNAs play specific biological roles in cancer migration and development. Yang has found that circAMOTL1L contributes to prostate cancer migration and EMT by sponging miR-193a-5p and affecting Pcdha level [15]. Bi reported that circRNA_102171 overexpression promoted papillary thyroid cancer migration via activation of Wnt/β-catenin pathway [22]. Additionally, circRNA_0023642 [23] and circRNA_103809 [24] have been reported to correlate with cancer migration. In the present study, we first confirmed that circFLNA was present in LSCC tissues and cell lines. As expected, circFLNA was upregulated in LSCC tissues and its expression level was correlated with lymph node metastasis. Thereafter, using a series of in vitro assays, we found that circFLNA acted as an important migration promotor in LSCC, suggesting that circFLNA may be a potential biomarker or a therapeutic target in LSCC.

FLNA is important for organogenesis during development owing to its ability to induce cell migration via its actin-binding properties [25]. It serves as a scaffold for over 90 binding partners and is involved in multiple cell functions, of which cell migration and adhesion are particularly critical [26]. Mechanistically, FLNA promotes cell adhesion and migration by directing β1 integrin to the site of cell attachment and through its correlation with vimentin [27, 28]. Recently, FLNA has been considered to be a tumor-promoting protein with an important role in tumor development and metastasis, including bladder cancer [16], lung cancer [29], melanoma tumor [30] and breast cancer [31]. Moreover, high level of FLNA implicates poor survival and drug resistance [32]. Therefore, FLNA may be a possible target for future therapies. In the present study, we found that overexpression of circFLNA in Hep2 cells increased the FLNA protein expression but did not affect the FLNA mRNA expression. miR-486-3p, as an important mediate, directly targeted the FLNA 3′UTR inhibiting FLNA expression that promoted LSCC migration. Meanwhile, LSCC tissues had a higher expression of FLNA mRNA than their corresponding non-tumorous tissues, as supported by the data from TCGA. A higher FLNA expression indicated poor prognosis in patients with LSCC. Based on these interesting findings, it was inferred that higher linear FLNA mRNA may directly inregulate the transcription level in LSCC; however, circFLNA indirectly enhances the level of FLNA protein in the post-transcriptional level. As previously mentioned, the expression of circRNA could not always be consistent with the level of which the circRNA-derived linear RNA [33]. Therefore, the underlying mechanisms of how and why both circFLNA and linear FLNA are upregulated in LSCC requires further investigation.

miRNA, as the most important non-coding RNA, post-transcriptionally regulates target gene protein expression through interaction with the 3′-UTR of their target genes in a sequence-specific base pairing manner [34]. Various aberrantly expressed miRNAs are associated with the progression and prognosis of LSCC. miR-370 targeted FoxM1 functions as a tumor suppressor in LSCC [35]; miR-1290 acts as a novel potential oncomiR in LSCC [36]; miR-1297 mediates PTEN expression and contributes to LSCC cell progression [37]. circRNAs contain one or more types of miRNA binding sites, and the association between miRNA and disease indicates that circRNAs may play a regulatory role by sponging miRNAs. Increasing evidence suggests that circRNAs mainly function in forming circRNA-miRNA-mRNA axis to play its biological effect in gene regulation. For example, circMTO1 acts as the sponge of miR-9 to suppress hepatocellular carcinoma progression [38]; circTCF25 serves a the regulatory role on the pathway in bladder carcinoma by binding to miR-103a [39]; circACTA2/miR-548-5p axis acts as a novel regulatory mechanism in smooth muscle alpha-actin expression [40]. In the present study, we confirmed that circFLNA could sponge miR-486-3p and prevent miR-486-3p from binding FLNA 3′UTR in LSCC cells. The results also indicated that miR-486-3p reduced LSCC cell migration by negative moderation of FLNA protein level. Interestingly, miR-486-3p is not significant different between LSCC tissues and adjacent normal tissues. It is suggested that circFLNA only contributes to miR-486-3p functions but not its expression. These findings suggest that miR-486-3p plays a critical role in LSCC migration.

## Conclusions

In conclusion, we showed that circFLNA was upregulated in LSCC tissues, and it can efficiently sponged miR-486-3p to inhibit FLNA expression. We also demonstrated that overexpression of circFLNA can effectively promoted migration of LSCC cells by targeting miR-486-3p/FLNA axis. Our findings provide a new therapeutic target for the treatment of LSCC.

## Data Availability

Not applicable.

## Abbreviations

LSCC: laryngeal squamous cell carcinoma
FLNA: filamin A (FLNA)
MMP2: matrix metalloproteinase-2 MMP2
circRNAs: circular RNAs
RT-qPCR: reverse transcription-quantitative polymerase chain reaction
MKL1: megakaryoblastic leukemia 1
